# "I am pregnant and my husband has diabetes. Is there a risk for my child?" A qualitative study of questions asked by email about the role of genetic susceptibility to diabetes

**DOI:** 10.1186/1471-2458-10-688

**Published:** 2010-11-10

**Authors:** Suzanne CM van Esch, Martina C Cornel, Frank J Snoek

**Affiliations:** 1 Department of Medical Psychology, VU University Medical Center, Amsterdam, The Netherlands; 2 Department of Clinical Genetics, VU University Medical Center, Amsterdam, The Netherlands; 3 EMGO Institute for Health and Care Research, VU University Medical Center, Amsterdam, The Netherlands

## Abstract

**Background:**

Diabetes Mellitus is a global health problem. Scientific knowledge on the genetics of diabetes is expanding and is more and more utilised in clinical practice and primary prevention strategies. Health consumers have become increasingly interested in genetic information. In the Netherlands, the *National Genetic Research and Information Center *provides online information about the genetics of diabetes and thereby offers website visitors the opportunity to ask a question per email. The current study aims at exploring people's need of (additional) information about the role of inheritance in diabetes. Results may help to tailor existing clinical and public (online) genetic information to the needs of an increasing population at risk for diabetes.

**Methods:**

A data base with emailed questions about diabetes and inheritance (n = 172) is used in a secondary content analysis. Questions are posted in 2005-2009 via a website providing information about more than 600 inheritable disorders, including all diabetes subtypes. Queries submitted were classified by contents as well as persons' demographic profiles.

**Results:**

Questions were received by diabetes patients (49%), relatives (30%), and partners (21%). Questioners were relatively young (54.8% ≤ 30 years) and predominantly female (83%). Most queries related to type 1 diabetes and concerned topics related to (future) pregnancy and family planning. Questioners mainly asked for risk estimation, but also clarifying information (about genetics of diabetes in general) and advice (mostly related to family planning) was requested. Preventive advice to reduce own diabetes risk was hardly sought.

**Conclusions:**

Genetic information on diabetes provided by professionals or public health initiatives should address patients, as well as relatives and partners. In particular women are receptive to genetic information; they worry about the diabetes related health of (future) offspring. It seems important that information on the contribution of genetics to type 1 diabetes is more readily available. Considering the high prevalence of type 2 diabetes with strong evidence for a genetic predisposition, more effort seems needed to promote awareness around familial clustering and primary prevention.

## Background

Diabetes Mellitus (a group of disorders characterised by abnormal high blood glucose levels) is a growing health problem [1]. In the last decade, scientific knowledge on the genetics of diabetes is expanding and has resulted in clinical application of genetic testing in the case of Maturity-Onset Diabetes of the Young (MODY) and Maternally Inherited Diabetes and Deafness (MIDD) [2]. The understanding of genetic variation predisposing to type 1 diabetes (T1DM) [3], Latent Auto-immune Diabetes in Adults (LADA) [4]), type 2 diabetes (T2DM) [5]), and gestational diabetes (GDM) [6]) is evolving. In the case of T2DM and GDM, family history is used as a marker for genetic susceptibility and a potential risk stratification tool in preventive activities [7-9].

Health consumers have become increasingly interested in genetic information [10,11]. This increasing interest is coupled with a growing trend in consumer uses of the Internet for health-related purposes. Statistics reveal that eight in ten American internet users (approximately 113 million adults) searched online for health information in 2006 [12]. In the Netherlands, about 93% of the population has access to Internet. Of all inhabitants using the Internet, 54% looked for information about health and medicines at least once in three months in 2009 [13].

While consumers recognise great potential in the Internet for health communication on human genetics [14], health professionals and genetic experts acknowledge that the translation of genomic information will be a challenge [15]. Information has to be adjusted to the (genetic) literacy levels of target audiences [16] and has to serve the public's genetic information needs [17]. Since the growing popularity of Internet use, indeed there are a lot of studies evaluating genetic web content [18,19], readability [20], and effect on behavioural outcomes [10]. In addition, the current study was designed to get insight into the public's interest, perception and information need about the genetics of diabetes.

In the Netherlands, the *National Genetic Research and Information Center *provides online information about the genetics of diabetes (all subtypes). Thereby, the Center offers website visitors the opportunity to ask a question per email. We used this data base with emailed questions to gain insight into people's need of (additional) information about the role of inheritance in diabetes. After all, depending on whether or not visitors read the provided information, the emailed questions reflect their information needs, unaddressed issues, areas of uncertainty or difficult to understand concepts.

Our research interest is in developing a profile of questioners, as well as the discovery of themes and tendencies in the emailed questions. Findings from this study will contribute to better understanding of specific information needs of online consumers about genetics and diabetes. The results may help to tailor existing clinical and public (online) health information to the needs of an increasing population at risk for diabetes [21,22].

## Methods

### Data source

The *National Genetic Research and Information Center *in the Netherlands provides online information about more than 600 inheritable disorders, including all diabetes subtypes http://www.erfelijkheid.nl/zena/diabe.php. Web statistics indicate that the general number of website visitors is reaching 2,2 million per year. Almost 9,000 visitors searched specifically for information on 'diabetes and inheritance' in 2009.

For each diabetes subtype, information about the pathophysiology, diagnosis, treatment, prevalence, and genetics is provided. Multifactorial-, monogenetic-, or mitochondrial inheritance is explained, and risk estimations are given for first- and second degree relatives of T1DM, T2DM, and MIDD patients. Links to other informative websites are given. Apart from reading the information on the website, visitors are offered the possibility to submit a question per e-mail. Three staff members, who are all educated in genetics and inheritance, answer the questions within three days. This helpdesk team refers to professionals in the field (e.g. clinical genetic centers, diabetes specialists or general practitioners) in case they are unable to answer the question. When posting a question on the website, visitors are invited to disclose information (optional) about their gender, age, and whether their interest is personal or professional, for the purpose of evaluation.

Since January 2005, the *National Genetic Research and Information Center *has systematically registered personal queries from website visitors. This data base with emailed questions is designed for administrative purposes, as well as monitoring the quality of the web content. For the current study, the Center handed over data concerning 'diabetes and inheritance' to the researchers and consented with the research objectives and methods. In view of the observational and non-invasive nature, this study is not subject to the Dutch Medical Research Involving Human Subjects Act. The researchers followed the rules defined in the Dutch Code of Conduct for Medical Research, in which a specific code for adequate secondary use of data is defined.

### Study sample

Data were derived from a sample of 265 e-mailed questions related to diabetes and inheritance (administered between January 2005 and November 2009). The *National Genetic Research and Information Center *assigned an identification number (#) to each email. The researchers received the emails without name and email address, to protect confidentiality of participants. A list with identification numbers and corresponding information about questioners' gender, age, and personal versus professional interest was enclosed.

It is not possible to ask informed consent of participants in secondary analyses, and we therefore excluded all questioners (n = 11) that opted not to provide any personal information. In addition, two exclusion criteria were applied. Fifty-eight e-mails did not relate to genetics and inheritance, but concerned diabetes (treatment) in general. Secondly, in this study we were primarily interested in (additional) information needs of 'private' health consumers, aiming at tailoring (online) information about diabetes and genetics. Thirty-eight questions were asked by students and health care professionals, and therefore were excluded from the sample. From the 158 e-mails left, fourteen contained two questions. In total, 172 queries were included in this study.

### Data analysis

In this study, we used secondary content analysis. The advantage associated with secondary data analysis is its convenience and cost-effectiveness [23]. We adopted an iterative and inductive approach which is argued to be applicable in computer-mediated convenience samples [24]. Two researchers (SvE and research assistant) double-coded all email questions using qualitative data indexing software (Kwalitan 5.0 [25]). Emerging themes and tendencies were identified and categorised; ambiguities were resolved and categories were reduced to major themes in discussion with two senior researchers and re-reading the emails [26].

After qualitative classification, data were quantified in order to develop participants' profile (by age, gender and family status) and observe the distribution of coding labels within the emerged categories ('type of diabetes inquired', 'topics inquired', 'expressed worry', and 'type of information requested'). Illustrative quotes are presented as summaries of the questions' quintessence, paraphrasing the original Dutch formulation as much as possible (however, sometimes with minor alteration to respect subjects' confidentiality). Participants' identification number (#), gender, age in years, and family status are included after each quote to help the reader identify the background of its source.

## Results

### Questioners' profile and type of diabetes inquired

As shown in Table 1, it appeared that most people asking questions via the website were relatively young; sixty-eight persons (54.8%) were ≤ 30 years. Mainly women inquired the role of inheritance in diabetes (n = 131; 82.7%). Nearly half of the questions were asked by diabetes patients (n = 77; 48.9%); almost one third by relatives (n = 47; 29.6%) and the remaining by partners of diabetes patients (n = 34; 21.5%).

**Table 1:** Questioners' profile, type of diabetes inquired and information provided about family history (n = 158)

Questioners' and questions' characteristics	N (%)
Age in years	
< 20	19 (12.1)
21-30	67 (42.7)
31-40	40 (25.0)
> 41	32 (20.2)
Gender	
Female	131 (82.7)
Male	27 (17.3)
Family status	
Patient	77 (48.9)
Relative	47 (29.6)
Partner	34 (21.5)
Type of diabetes inquired	
Type 1 diabetes	59 (37.3)
Type 2 diabetes	15 (9.5)
Type 1 and type 2 diabetes	13 (8.2)
Gestational diabetes	13 (8.2)
MODY/MIDD/LADA	8 (5.0)
Diabetes insipidus*	6 (3.8)
Diabetes type not specified	44 (27.9)
Question lacks well-defined information about family history	86 (54.6)

* Although the name is rather similar, diabetes insipidus is a different clinical entity (left out of the scope of this paper).

Most questions concerned T1DM (n = 59; 37.3%). Relatively few questions referred to T2DM (n = 15; 9.5%) and GDM (n = 13; 8.2%). Thirteen participants inquired about T1DM as well as T2DM (8.2%), since they seemed confused about the presence of both diabetes subtypes in their family. A T1DM patient for example asked: "I am pregnant. Both my parents have type 2 diabetes. Is my baby at increased risk for type 1 or type 2 diabetes?" [#3129 Female, 27y, Patient]. Other types of diabetes, like MODY, MIDD or LADA, were rarely inquired (n = 8; 5.0%).

Notably, in forty-four questions (27.9%) the type of diabetes was not specified, for example: "Has diabetes in men consequences for offspring?" [#2097 Female, 31y, Partner], or "I have diabetes. My partner's father and grandfather also have diabetes. We are thinking about having children. Is diabetes inheritable and if yes, what is the diabetes risk of our children?" [#2088 Female, 18y, Patient]. Additionally, slightly more than half of the questions (n = 86; 54.6%) did not contain well-defined family information, for instance: "Five persons in my family have diabetes. Does this have to do with inheritance?" [#3104 Female, 65y, Relative].

### Topics that people inquire about

Table 2 displays the topics of interest. Eighty-four participants (48.8%) inquired about genetics and inheritance in relation to reproduction. More than half of these questions specifically referred to a (future) pregnancy. Evidently, the period of pregnancy brings up worries about consequences for the questioners' health as well as the health of the foetus, for instance: "I am 13 weeks pregnant and have type 1 diabetes. My blood glucose levels are very unstable. I am worried about my health and possible consequences for my baby." [#5012 Female, 30y, Patient], or in case of GDM: "I have gestational diabetes. What are the risks for the baby during pregnancy and what is the risk for the child later in life?" [#0014 Female, 26y, Patient]. Even regarding to late onset T2DM people seem to be worried: "I am six weeks pregnant. My husband is a type 2 diabetes patient. Is there a risk for my child?" [#1048 Female, 31y, Partner]. The preconception phase was mostly inquired by T1DM patients: "I have so many questions. I have type 1 diabetes and want to get pregnant. Is that possible? What are the risks for me, the pregnancy and the baby?" [#3131 Female, 36y, Patient]. Besides pregnancy, 'family planning' was often explicitly mentioned as a reason for asking the question. A few questioners were in serious doubt about having offspring, because of the (sometimes high) prevalence of diabetes in their family. As one patient stated: "Three persons in my family, including myself, have type 1 diabetes. A fourth family member is diagnosed with LADA. What is the chance my future children will develop diabetes? I don't know whether I want to have children, if they would be at really high risk." [#5055 Female, 24y, Patient].

**Table 2:** Topics that people inquire about, expressed worry, and type of information requested

	N (%)
Topics inquired	
Genetics and inheritance in relation to reproduction	84 (48.8)
Genetics and inheritance in general	64 (37.2)
(New) technologies: genetic testing, gene therapy	24 (14.0)
Expressed worry	
Worry about offspring's diabetes risk	78 (45.3)
Worry about own diabetes (risk)	58 (33.7)
Not explicitly mentioned	36 (20.9)
Type of information requested	
Risk estimation	69 (40.1)
Asking for an explanation/clarification/verification	42 (24.4)
Looking for advice	38 (22.1)
Asking (specified) information	23 (13.4)

More than one third of the e-mails (n = 64; 37.2%) concerned the genetics of diabetes 'in general'. Most questioners inquired about the role of inheritance in their family, for instance: "Two of my kids have type 1 diabetes. Both my parents have type 2 diabetes. Since type 2 diabetes is not inheritable, why do my children have type 1 diabetes?" [#1025 Female, 28y, Relative]. Some people are specifically interested in genes: "I have type 1 diabetes, celiac disease and epilepsy. Which gene defects are causing these diseases? Are these genes related?" [#1018 Male, 30y, Patient].

A small amount of e-mails (n = 24; 14.0%) concerned queries about (new) technologies like genetic testing, genetic therapy and progresses in the scientific field, for example: "Which medical center in the Netherlands performs genetic tests for MODY? Which qualifications for testing are required?" [#3105 Male, 35y, Patient]. A questioner with high expectations asked: "I have type 2 diabetes. When will gene therapy be available?" [#1039 Male, 28y, Patient].

### Expressed worry

It appeared that almost half of the queries received (n = 78; 45.3%) were related to worries about (future) offspring's diabetes risk. One third (n = 58; 33.7%) referred to the questioner's own diabetes related health or diabetes risk. Noteworthy, some people inquiring offspring's diabetes risk seem not worried or even aware of their own possibly increased diabetes risk, for instance: "My partner has type 1 diabetes. My father, and possibly my mother-in-law, had type 2 diabetes. What is the chance my child will develop type 1 diabetes?" [#4027 Female, 36y, Partner]. Or: "My uncle has diabetes. Is it possible this disease is inheritable for my future child?" [#1040 Female, 25y, Relative]. Not all questioners did explicitly express worry in their e-mail.

### Type of information requested

Table 2 summarises the type of information that website visitors requested. Most e-mail questions (n = 69; 40.1%) pertained a request for (personalised) risk information. On the website, risk estimations for first- and second degree relatives of T1DM, T2DM, and MIDD patients are described. In theory, questions like: "My partner has type 1 diabetes. What is the risk my future child will develop diabetes?" [#0106 Female, 29y, Partner] could be answered by reading this information. Evidently, in families with complex family history (different types of diabetes and/or diabetes running through several generations), the provided risk information might be difficult to apply. For instance, "A relative of mine has type 1 diabetes. In my wife's family type 1 as well as type 2 diabetes is prevalent. Is it possible to estimate whether my children are at increased risk for type 1 or type 2 diabetes?" [#3154 Male, 28y, Relative].

In almost one quarter of the e-mails (n = 42; 24.4%), the questioner requested an explanation, clarification or verification. For example, people inquired about the (genetic) co-occurrence of different types of diabetes in their family: "Are gestational diabetes and diabetes insipidus genetically related?" [#3134 Female, 46y, Relative]. Some wanted to verify or validate information or ideas: "I have type 1 diabetes. My father, my aunt and grandmother also have type 1 diabetes. I was told it's a coincidence. Is that true?" [#2079 Female, 25y, Patient], or "I have type 1 diabetes. Several family members have type 2 diabetes. Is it possible I inherited my diabetes?" [#2066 Female, 16y, Patient].

Thirty-eight participants (22.1%) expressed a wish to obtain preventive and/or therapeutic advice, either with regard to one's own health or the diabetes related health of offspring. For example: "We are thinking about a pregnancy. My partner has type 1 diabetes. In my family, some relatives have type 2 diabetes. What precautionary measures do we have to take to get a healthy baby?" [#1016 Female, 29y, Partner]. Preventive advice to reduce own T2DM risk was hardly sought. As regards, only three questioners referred to the multifactorial aetiology of T2DM: "My father has type 2 diabetes. How can we prevent developing diabetes?" [#0012 Female, 40y, Relative].

Finally, in some e-mails (n = 23; 13.4%) people included a clear request for specific information, like a MODY patient stated: "I am looking for information. My son and I are diagnosed with MODY, caused by a heterozygote mutation." [#3138 Female, 30y, Patient]. Or a patient was interested in scientific progresses: "How far are developments in the field of stem cell transplantation or other possible solutions to cure type 1 diabetes?" [#5016 Male, 41y, Patient].

### Information need

To get a clearer view of questioners' information needs, we combined the topics they inquired about with the type of information they requested. It appeared that questioners inquiring about genetics in relation to reproduction most of the time were in need of risk information (n = 40; 47.7%) or advice (n = 30; 35.8%). The majority of queries concerning genetics and inheritance of diabetes in general also contained a need for risk information (n = 28; 43.8%) and in almost an equal number of cases a request for explanation or verification (n = 26; 40.6%). People submitting an e-mail about (new) technologies in the genetic field mostly demanded specified information (n = 14; 58.3%) (see Table 3).

**Table 3:** Topics inquired about related to the type of information requested*

	Topics inquired about		
	
Type of information requested	Reproduction (n = 84)	Genetics in general (n = 64)	(New) technologies (n = 24)
**Risk estimation**	47.7	43.8	4.2
**Explanation/verification**	15.5	40.6	12.5
**Advice**	35.8	3.1	25.0
**(Specific) information**	0.0	12.5	58.3

* Data are percentages

## Discussion

Based on e-mails received by the Dutch *National Genetic Research and Information Center*, it appears that people in need of (additional) online information about diabetes and inheritance are relatively young and predominantly female. This is in line with previous research indicating that younger Internet users and women are most likely to search for online genetic information [27,28]. Yet, the online population is expanding and becoming more representative in terms of race, age, income, and educational attainment [29,30]. Interestingly our data suggest that besides patients with diabetes, relatives and partners seem interested in the topic of inheritance. This is in contrast to earlier reports in the field of oncology where partners and relatives are described as potential 'blockers' of genetic information in families [31]. This difference may at last partly be related to the disease at stake and warrants further investigation.

Although only accounting for 5-10% of the overall prevalence of diabetes, most questions concerned T1DM. Apparently T1DM is assumed to be genetic, probably because of its juvenile onset. More interest could be expected regarding the genetic subtypes of diabetes, MODY and MIDD, although they are rare and treated in specialty clinics where genetic information may be readily available [2]. Considering the high prevalence of T2DM (~90% of all diagnosed cases) and GDM (2-5% of all pregnancies, with higher prevalence in some ethnic/racial groups [32]), the role of heredity in these subtypes appears to be under appreciated. Possibly, the information need is low due to underestimation of the seriousness of the condition [33] or limited awareness about the role of genetics and shared environment in the aetiology [34,35]. Recent trends indicating a growing awareness of family risk and worry about the development of T2DM in offspring [36,37] is not yet reflected in our results. Possibly, people found genetic information about T2DM, GDM or MODY/MIDD/LADA subtypes on other websites than the one we studied. Research however reveals that most online health information seekers start their session at a search engine [12] and using Google, the first (and almost only) hit when searching for 'diabetes and inheritance' in Dutch refers to the website of the *National Genetic Research and Information Center*. Moreover, we found earlier that information on diabetes and inheritance provided by websites of renowned diabetes organisations is generally poor or lacking [18].

Finally, data reveal that the majority of queries concern topics related to (future) pregnancy and family planning. This finding is in line with the relatively young age of questioners and overrepresentation of women in our study. It is known that the phase of reproduction generates an active search for genetic information [28,38]. Women have been found to search for genetic information, because they worry about the health of their (future) offspring [39]. These results resemble our finding that in most queries worry about the diabetes related health of (future) offspring is expressed.

### Strengths and limitations

A strength of this study is that data were collected from a registry of people's search for information in a 'natural' setting as opposed to exploring beliefs and knowledge on genetics in a (high-risk) clinical setting or in general public using questionnaires [40]. We were able to explore questions based on individual perceptions and interests, described in people's own words.

However by utilising secondary data analysis, we were unable to further expand our understanding by posing additional questions for example related to the amount of visitors actually reading the information provided on the website, the degree of understanding, and perceived utility of the expert answers received [23]. On the other hand, it appeared that the available 172 queries generated a study sample that was rich enough to emerge categories reflecting interesting themes and tendencies to describe.

We are aware that people submitting e-mail questions via the Internet may represent a selective group (in our study: young, predominantly female Internet users) and we cannot exclude selection bias. Also, the study's generalizability is limited due to its reliance on questions gathered by only one web based supplier of genetic information. It would be interesting to expand our study using other interactive websites and other countries to provide insight in other settings and cultures.

### Practical implications

In earlier research, it appeared that physicians are the preferred first source of health information for 50% of Americans. Yet only 11% report their physician as the first line of inquiry, as compared with 49% who report that the Internet is their first source [41]. Consequently, the delivery of genetic information on diabetes is important in clinical practice as well as in (web based) public health initiatives.

Diabetes professionals might adjust their information after discussing clients' information needs, family situation and risk perception. It is important to notice that some recipients will be in need of personalised risk information, while others prefer clarifying information or advice. Public health initiatives, including web based strategies, can add to the health education of people about genetic backgrounds of common diseases, and provide general risk information as well as preventive messages. Also, information on scientific progress and new technologies in the field of genetics may fulfil the need of a small, but highly interested public.

In addition of public information, individuals may wish to receive personalised (risk) information or advice. Utilising an email approach often requires more detailed information from the person than currently provided. As an alternative, clinicians and public health providers could compile a list of frequently asked questions (and answers) about diabetes and inheritance and incorporate it into (web based) diabetes family education.

## Conclusion

Utilising genetic information requires a well-considered strategy. Our study suggests that patients, in particular women, but also relatives and partners, are in need of information on the genetics of diabetes. Preventive advice to reduce own diabetes risk was hardly sought. Considering the high prevalence of T2DM and GDM, more effort seems needed to explain the multifactorial aetiology (and with it, the risk of familial clustering). Opportunities to delay or prevent T2DM and GDM onset by adopting a healthy lifestyle [42,43] should be emphasised. To optimise health behaviour, these efforts should take public perceptions about inherited predisposition and primary prevention into account [44,45]. Findings from this study underscore the importance of further exploring the genetic information needs of people with diabetes of all types. At least it seems important that information on the contribution of genetics to T1DM is more readily available.

## Competing interests

The authors declare that they have no competing interests.

## Authors' contributions

SvE collected data, performed the analyses and drafted the manuscript. Both MC and FS contributed to the interpretation of data, reviewed versions of the article and suggested revisions. All authors read and approved the final manuscript.

## Pre-publication history

The pre-publication history for this paper can be accessed here:

http://www.biomedcentral.com/1471-2458/10/688/prepub
